# Evaluation of pre-workout and recovery formulations on body composition and performance after a 6-week high-intensity training program

**DOI:** 10.3389/fnut.2022.1016310

**Published:** 2022-11-02

**Authors:** Hannah E. Cabre, Amanda N. Gordon, Noah D. Patterson, Abbie E. Smith-Ryan

**Affiliations:** ^1^Applied Physiology Laboratory, Department of Exercise and Sport Science, University of North Carolina at Chapel Hill, Chapel Hill, NC, United States; ^2^Human Movement Science Curriculum, Department of Health Science-UNC School of Medicine, University of North Carolina at Chapel Hill, Chapel Hill, NC, United States; ^3^Department of Nutrition, Gillings School of Global Public Health, University of North Carolina at Chapel Hill, Chapel Hill, NC, United States

**Keywords:** interval exercise, dietary supplement, resistance training, untrained, sex differences, protein, ergogenic aid

## Abstract

**Introduction:**

Activities such as high-intensity resistance training (HIRT) and high-intensity interval training (HIIT) may be more time-efficient modes to stimulate rapid changes in performance and body composition. There is little research evaluating the combined effects of HIRT and HIIT on body composition and strength, particularly when paired with nutritional supplementation.

**Purpose:**

To evaluate the chronic effects of pre- and post-workout supplementation on body composition and strength, and to understand sex-specific responses.

**Materials and methods:**

64 untrained males (*n* = 23) and females (*n* = 41) (mean ± standard deviation; age: 33.2 ± 10.0 years; %fat: 31.6 ± 7.4%) were randomized to either (1) pre-post supplementation [SUP (*n* = 25); pre = multi-ingredient caffeine/HMB/vit D; post = whey protein/carbohydrates/glucosamine/vitamins], (2) placebo [PL (*n* = 24); non-caloric], or (3) control [CON (*n* = 15)]. All participants completed one repetition max (1RM) strength testing for leg press and bench press at baseline and week 6. Estimates of fat mass (FM) and lean mass (LM) were measured *via* dual energy x-ray absorptiometry. Participants in the SUP or PL group completed a 6-week supervised exercise intervention consisting of a full-body HIRT workout (3 × 6–8 reps) followed by a HIIT treadmill run (6 × 1 min run: 1 min rest) twice per week. Outcomes were evaluated by separate repeated measure ANOVAs (2 × 3).

**Results:**

There were no differences in FM between groups or sex (*p* = 0.133–0.851). LM increased from baseline to post-testing for all groups [Mean difference [MD(Post-Pre) ± Standard Error (SE) = 0.78 ± 0.12 kg; *p* < 0.001]. While not significant (*p* = 0.081), SUP gained more LM compared to PL [MD(SUP-PL) ± SE = 3.5 ± 3.3 kg] and CON [MD(SUP-CON) ± SE = 5.2 ± 3.8 kg]. LM increased over time for both males (0.84 ± 0.24 kg; *p* = 0.003) and females (0.73 ± 0.14 kg; *p* < 0.001). The SUP group resulted in a significant increase in 1RM leg press compared to the CON group (89.9 ± 30.8 kg; *p* = 0.015), with no significant differences compared to PL (*p* = 0.409). The SUP group had greater increases in 1RM bench press compared to the CON group (9.8 ± 1.8 kg; *p* < 0.001), with no significant differences compared to PL (*p* = 0.99). Both sexes increased upper- (5.5 ± 0.7 kg; *p* < 0.001) and lower-body strength (69.8 ± 4.5 kg *p* < 0.001) with training.

**Conclusion:**

Nutrient supplementation timing appears to augment body composition changes and strength compared to control. Pre-/post-nutrient timing may support greater increases in LM and lower- and upper-body strength in both men and women.

**Clinical trial registration:**

[https://clinicaltrials.gov/ct2/show/NCT04230824?cond=NCT04230824&draw=2&rank=1], identifier [NCT04230824].

## Introduction

In light of the COVID-19 pandemic, working adults reported having less time due to greater work and home-life demands, highlighting that the current physical activity guidelines may be unattainable (1). As such, 80% of American adults do not meet the recommended physical activity guidelines, with lack of motivation and time being the most commonly cited barriers (2, 3), further emphasizing the importance of identifying practical and feasible exercise and nutrition strategies. Activities such as high-intensity resistance training (HIRT) and high-intensity interval training (HIIT) have emerged as more time-efficient modes to stimulate rapid changes in cardiometabolic health (4). These exercise strategies may improve engagement, while eliciting similar improvements in body composition compared to traditional exercises (5, 6), particularly when paired with nutritional support (5, 7, 8).

Broadly, HIRT requires participants to lift a heavy load with short recovery between sets reducing the total training time (9, 10). Compared to traditional resistance training, HIRT has been shown to significantly increase resting energy expenditure after exercise, and may improve fat oxidation (9). These adaptations may be beneficial for strength and body composition, particularly increases lean mass (LM) and decreases in fat mass (FM) (9, 10). Furthermore, HIRT mimics aerobic HIIT training, alternating repeated bouts of exercise at near maximal intensity (∼90%) interspersed with periods of rest or low intensity exercise (11). Prior research on HIIT training has largely focused on the rapid aerobic and metabolic adaptations that occur, yet more recent research has demonstrated advantageous improvements in LM and muscle size in as little as three weeks (6). While concurrent aerobic exercise and resistance training typically result in decreased hypertrophy and strength (12), HIIT may be an effective aerobic method for maintaining strength and LM (13). However, there is little research evaluating the combined effects of HIRT and HIIT on body composition and strength, or the additive effects with planned nutritional supplementation around exercise (14).

Nutrient timing may augment adaptations from HIRT and HIIT by enhancing energy availability and the adaptive responses to exercise (14). While there is conflicting information surrounding the impact of nutritional composition and timing on exercise (7, 15–18), data collectively supports nutrient consumption surrounding exercise augments exercise adaptations compared to withholding nutrients (17). Specifically, existing data support a potential synergistic effect of nutrient timing and HIRT + HIIT, respectively. When protein consumption prior to or post-HIRT was compared to no nutrient consumption in women, both groups consuming protein demonstrated greater increases in LM and strength (7). An investigation evaluating a multi-ingredient pre-workout supplement (caffeine, creatine, and amino acids) consumed prior to HIIT resulted in significant improvements in LM and anaerobic capacity (19). Taken together, nutrients paired with high-intensity exercise may act synergistically to promote greater changes in body composition and strength. However, the timing of nutrients is variable in the literature with most studies focusing on either pre- or post-nutrient consumption separately rather than a combinatory approach (14).

Other nutritional ingredients have gained popularity such as β-hydroxy-β-methyl butyrate (HMB), vitamin D, and fish oil due to their positive effect on tissue repair (20) and inflammation (21, 22), possibly leading to improved recovery from intense exercise and maintenance of LM. However, whether these results translate to concurrent HIRT + HIIT training is unclear. Furthermore, sex-based differences exist in muscle and mitochondrial biogenesis in response to interval training (23). These differences are important considerations when evaluating body composition and strength outcomes, but investigations on the sex effects of exercise + nutrition is nearly nonexistent. Therefore, the purpose of this study aimed to evaluate the chronic effect of pre-and post-workout supplementation combined with a concurrent HIRT + HIIT exercise intervention, compared to placebo and control, on body composition, performance (VO_2_max, 1RM strength, counter movement jump), and recovery (creatine kinase, isoprostanes) in inactive males and females; an exploratory aim was to investigate sex differences in body composition and performance. It was hypothesized that the nutrient timing would lead to greater improvements in body composition, performance, and recovery compared to placebo, and control.

## Materials and methods

### Subjects

**FIGURE 1:**
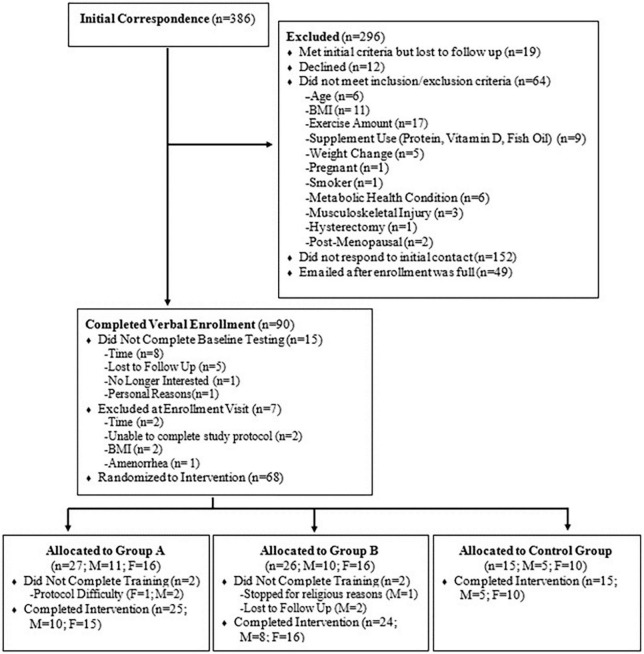
CONSORT recruitment.

Sixty-four healthy, untrained males (*n* = 23) and females (*n* = 41) (mean ± standard deviation (SD); age: 33.2 ± 10.0 years, height: 169.8 ± 10.2 cm, weight: 73.6 ± 15.5 kg, BMI: 25.2 ± 3.7 kg/m^2^) (Table 1) were recruited to participate in this study. Full CONSORT information is reported in Figure 1. All participants were healthy, non-smokers, between the ages of 18–52 years, with a BMI between 18.5 and 35 kg⋅m^–2^, and did not participate in more than 3 h per week of exercise, resistance training, and/or interval training. Participants were not consistently consuming any prescription medications for blood pressure or supplements that would influence study outcomes such as beta-alanine, creatine, beta-hydroxy-beta-methylbutyrate, carnosine, vitamin D (>1,000 IU/day), protein powder, or fish oil (>1,000 mg/day). Participants were excluded if they had a weight gain or loss of 3.6 kg within the 30 days prior to enrollment, were pregnant or planned to become pregnant as confirmed by urine HCG, or were sensitive/allergic to any of the ingredients in the test product (Table 2). A health history questionnaire was used to confirm inclusion/exclusion criteria. All methodology was approved by the University’s Institutional Review Board, and all participants provided verbal and written informed consent prior to participation.

**TABLE 1:** Participant characteristics presented as mean ± standard deviation.

Variable	Supplement group (*n* = 25; *M* = 11; *F* = 16)	Placebo group (*n* = 24; *M* = 10; *F* = 16)	Control group (*n* = 15; *M* = 5; *F* = 10)
Age (years)	31.3 ± 9.7	35.6 ± 10.2	32.9 ± 10.1
Height (cm)	171.7 ± 9.3	169.0 ± 11.0	167.8 ± 10.2
Weight (kg)	77.2 ± 16.2	71.3 ± 12.3	71.4 ± 18.7
BMI (kg/m^2^)	26.1 ± 4.4	24.8 ± 2.2	24.4 ± 4.3
Average total calories (kcal/day)	1889.1 ± 558.6	2079.3 ± 437.6	2012.0 ± 498.3
Average protein (g/day)	85.0 ± 26.1	87.8 ± 25.1	95.2 ± 29.7
Average carbohydrate (g/day)	209.1 ± 97.5	228.1 ± 50.9	208.5 ± 78.3
Average fat (g/day)	75.3 ± 30.8	86.8 ± 22.1	82.0 ± 27.5
Relative protein intake (g/kg/day)	1.1 ± 0.3	1.3 ± 0.3	1.4 ± 0.4
Relative carbohydrate intake (g/kg/day)	2.8 ± 0.9	3.3 ± 0.8	3.2 ± 1.4
Relative fat intake (g/kg/day)	1.0 ± 0.4	1.24 ± 0.3	1.18 ± 0.4
Average HIRT training volume (kg)	67303.1 ± 29428.9	60483.4 ± 25350.0	–
Average HIIT training volume (km)	12.0 ± 3.2	12.4 ± 3.0	–

M, male; F, female; BMI, body mass index; kcal, calories; g, gram; kg, kilogram; HIRT, high intensity resistance training; HIIT, high intensity interval training; km, kilometers. There were no significant differences between groups for these outcomes (*p* > 0.05).

**TABLE 2:** Multi-ingredient active supplement descriptions.

Active pre-workout	

Ingredient	Amount
Caffeine	50 mg
Choline bitartrate	550 mg (226 mg choline)
Carbohydrate (palatinose)	5 g
β-hydroxy-β-methylbutyrate (HMB)	1.5 g
Vitamin D_3_	500 IU
**Active post-workout**	
Whey protein	15 g
Caseinate protein	5 g
Carbohydrates	20 g (10 g palatinose and 10 g corn starch)
Vitamin C	200 mg
D-alpha tocopherol	45 IU
Vitamin D_3_	1,000 IU
Glucosamine	1.5 g

Mg, milligrams; g, grams; IU, international units.

### Experimental design

**FIGURE 2:**
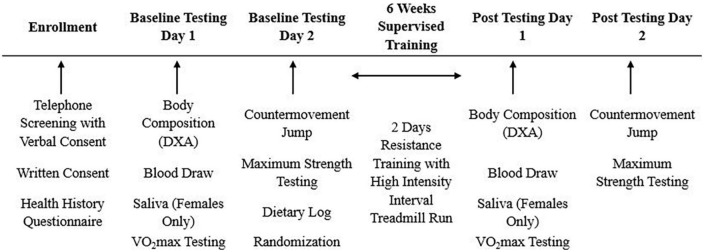
Experimental design.

This study was a randomized, double-blind, placebo-controlled trial. Participants were asked to abstain from food and caloric beverages (12 h), caffeine (12 h), alcohol (24 h), and physical activity (24 h) prior to baseline and post-testing sessions. At baseline, participants completed a maximal graded exercise test on a treadmill to volitional exhaustion, and a maximal strength protocol to determine the appropriate intensities for the exercise training. Body composition measures, blood markers of muscle damage/recovery, and countermovement jumps were also evaluated. Participants were then randomized in a 2:2:1 fashion to a (1) active ingredient supplement (SUP; exercise intervention with pre-post exercise supplementation), (2) placebo (PL; exercise intervention with non-caloric placebo provided before and after exercise; Crystal Light), or (3) control group (CON; no exercise or treatment assigned). Participants in the SUP treatment or PL group completed a 6-week supervised exercise intervention consisting of a full-body high-intensity resistance training workout followed by a high-intensity interval treadmill run twice per week. All groups participated in post-testing session identical to baseline testing within 48–120 h after the final training session, refraining from exercise at least 24 h prior (Figure 2). The number of participants per day for the post-testing visits are as followed: 48 h *n* = 10; 72 h *n* = 13; 96 h *n* = 6; 120 h *n* = 19.

### Dietary intake

All participants were asked to complete a three-day food record (two weekdays and one weekend day) at baseline prior to training. The dietary information was entered into Food Processor (ESHA Research; Version 10) to account for nutrient intake including total calories (kcal/day), carbohydrates (g/day), protein (g/day), and fat (g/day). Participants were asked to maintain dietary habits for the duration of the study. Estimated daily caloric needs per participant were calculated using the Harris Benedict Equation with an activity factor of 1.375. The difference between estimated energy expenditure and actual dietary intake was calculated and stratified by above and below estimated total daily energy requirements, yet there were not significant differences in dietary intake.

### Body composition

Participant body composition was evaluated utilizing a whole-body DXA scan (GE Lunar iDXA, GE Medical Systems Ultrasound and Primary Care Diagnostics, Madison, WI, USA). Prior to each use, the device was calibrated according to the manufacturer’s guidelines. Each participant’s sex, birthdate, height, weight, and ethnicity were entered into the software (enCORE Software Version 16) prior to the scan. Participants wore loose athletic clothing and removed all metal and heavy plastic to reduce scan interference. Each participant was positioned supine in the center of the scanning table by a trained technician. Regions of interest were manually adjusted by the technician to determine lean mass (LM), fat mass (FM), and body fat percentage (%BF). DXA test-retest reliability from this laboratory for individuals of similar stature included intraclass correlation coefficient (ICC) = 0.99 and standard error of the measurement (SEM) = 1.07 kg for LM, ICC = 0.98 and SEM = 0.85 kg for FM, and ICC = 0.96 and SEM = 1.279% for %BF.

### Maximal oxygen consumption (VO_2_max)

To determine peak oxygen consumption (VO_2_max) to establish exercise intensity, all participants completed a graded exercise test to volitional exhaustion on a treadmill (Woodway Treadmill Woodway USA, Inc., Waukesha, WI). For males, following a 3-min warm up at 5.6 km/h, intensity was increased to 9.0 km/h and was then increased by 1.1 km/h every 3 min until 18.0 km/h. For females, following a 3-min warm up at 5.6 km/h, intensity was increased to 7.2 km/h and was then increased by 1.1 km/h every 3 min until 16.3 km/h. Breath-by-breath respiratory gases were analyzed with fifteen-second averages using indirect calorimetry (Parvo Medics TrueMax 2400^®^, Salt Lake City, UT); the three highest oxygen consumption values were averaged and recorded as VO_2_max (VO_2_max; ml⋅kg^–1^⋅min^–1^). The test was considered maximal if it met a minimum of two of the following criteria: a plateau in heart rate (HR) or was within 10% of age-predicted HRmax; a plateau in VO_2_ or no more than a 150 ml⋅min^–1^ increase; a respiratory exchange ratio value greater than 1.15 a.u. Peak speed was used to establish individual exercise intensity for the interval exercise training. Previous test-retest reliability for this VO_2_max protocol resulted in an ICC = 0.98 and SEM = 1.17 ml/kg/min, respectively.

### Strength testing

To determine one repetition maximum (1RM; kg) for leg press and bench press, each participant performed a set of 8–10 repetitions, with a weight that is approximately 50% of the anticipated 1RM as a warmup. The load was increased to 80% of the predicted 1RM, and participants were asked to perform of 4–6 repetitions. The weight was then increased to an estimated 1RM load, and the participants attempted a single repetition with the weight. After the completion of each successful 1RM attempt, the weight was increased until failure was reached, with 2–3 min of rest between each 1RM attempts. Leg press 1RM was determined first followed by 1RM for bench press. The leg press and bench press 1RM were used to estimate 75% to 85% of maximum load for the resistance training bouts. A systematic review on test-retest reliability for 1RM tests demonstrated an ICC = 0.97 and median coefficient of variation (CV) = 4.2% (24).

Multiple RM tests, specifically a 6RM, was used to predict participants’ 1RM on four different accessory exercises. These exercises included an overhead shoulder press, a bicep curl, an overhead tricep extension, and an alternating stationary lunge, all using dumbbells. The research staff determined what weight participants began with for each exercise, based on the prior training of the individual, aiming for 3–10 successful repetitions. Participants were allowed approximately 2 min of rest between each accessory exercise. The amount of weight used (rep weight) and the number of repetitions completed until fatigue (RTF) was put in the following equation to predict participants’ 1RM (25):


(1) $$1\,R\,M=\frac{r\,e\,p\,w\,e\,i\,g\,h\,t}{0.522+0.419\,e^{-0.055^{*}\,R\,T\,F}}$$

The projected 1RM value that was calculated from this equation was then used to estimate 75% to 85% of maximum load for the resistance training bouts.

### Counter movement jump

Participants completed three maximal countermovement vertical jumps using a Just Jump™ mat (Just Jump or Run, Probotics, Inc., Huntsville, AL, USA), each separated by 30 s of rest. Participants were positioned with feet shoulder-width apart and instructed to jump vertically, as high as possible, and return to the same position with both feet landing at the same time. Jump height (cm) was calculated automatically using the flight time from when the participant’s feet left the mat until landing. The greatest jump height was determined as the CMJ. Test-retest reliability for 1RM tests demonstrated an ICC = 0.93 and Cronbach’s alpha = 0.96 (26).

### Blood analytes

A 12 ml venous blood sample was obtained from the antecubital region of the arm at baseline and during post-testing. The blood samples were obtained at the post-testing visit within ± 2 h of the time of day of baseline blood sample collection. Blood was sampled to determine the concentration of creatine kinase and isoprostanes. Blood samples for isoprostanes were immediately centrifuged at 3,000 rpm at 5°C for 10 min. Aliquots of serum for the isoprostanes were frozen at −80^°^C for batch analysis and were analyzed using commercially available, enzyme-linked assays (8 isoprostane ELISA Kit; ab175819; abcam, Cambridge, MA, USA). Blood samples for creatine kinase were allowed to coagulate at room temperature for 30 min and then were immediately centrifuged at 3,000 rpm at 5°C for 10 min. Samples for creatine kinase and isoprostanes were analyzed by LabCorp (Burlington, NC, USA). The average coefficient of variation in creatine kinase between duplicates samples was 3.65%, while isoprostanes was 3.54%.

### Supplementation

Treatment randomization was assigned in a 2:2:1 group allocation for the SUP (Table 2 composition), PL, and CON, respectively, using Random Allocation Software (Sealed Envelope Software; Sealed Envelope Ltd., London, UK). Treatments were packaged and supplied in numerically labeled opaque containers by the Sponsor (Nu Skin, NSE Products, Inc., Provo, UT, USA) to maintain a double-blinded design. Participants were provided with their assigned treatment SUP pre-workout and post-workout, or non-caloric flavored powder blend PL (Crystal Light). Treatments were only consumed on training days (2 × per week). Participants were instructed to consume the pre-workout supplement with four- eight ounces of water 30 min prior to arriving for training visits. If participants did not consume the pre-workout supplement prior to the visit, they consumed the appropriate treatment at the laboratory and waited the 30 min before beginning the exercise training. The post-workout treatment was prepared with 4–8 ounces of water by research staff and was ingested by the participants in the laboratory within 15 min of cessation from exercise. Average group compliance (SUP *n* = 25; PL *n* = 24) was determined by dividing the total number of doses consumed by the total number of doses allotted. Compliance for the SUP group was 99.1%, and for the PL group was 98.8%.

### Exercise intervention

Participants engaged in a progressive, supervised six-week high-intensity resistance training program as previously described (7, 9). Training took place two days per week in the laboratory, with at least 24 h, but not more than 10 days, separating each training visit. The initial weight for the leg press and bench press at the first training session was set at 80% of the participants’ 1RM. The initial weight for the four accessory exercises was set at 75% of the participants’ projected 1RM. Heart rate was continuously monitored using a Polar Heart Rate monitor and participants reported perceived rate of exertion (Borg scale) after each exercise set. Resistance exercises were performed in the following order, under one-on-one supervision from laboratory staff: leg press, bench press, lunges, shoulder press, bicep curl, and triceps extension. Three sets of each exercise were completed for 6–8 repetitions, with 20–30 s rest between sets and 2:30 s rest in between each exercise. Load for each exercise was increased when participants successfully completed at least eight repetitions for each set, the weight was increased by 10% for lower body exercises and 5% for upper body exercises. Load was evaluated after each training session. Sessions were overseen and progressed by trained research staff. Following the resistance training, an interval exercise session occurred on the treadmill consisting of 5–6 bouts of 1-min of high-speed running at 90–100% peak speed during VO_2_max, interspersed with a 1-min rest/walk period. If participants were unable to run on the treadmill, the interval training was completed on a cycle ergometer with the wattage determined by target heart rate. Training volume for HIRT was determined by product of sets × repetitions completed × external load used. Training volume for HIIT was determined by converting the miles per hour to kilometers per hour and multiplying speed × bout × duration (time) of bout (Table 1).

### Statistical analysis

The Shapiro–Wilk test was used to determine if all data were normally distributed. Outliers were removed if the value was 3 standard deviations (SD) above or below the mean (*n* = 8 time points) (27). Baseline characteristics between groups were assessed with a one-way ANOVA. A series of 3 × 2 [group (SUP vs. PL vs. CON) × time (Baseline vs. Post)] repeated measures ANOVAs were used to evaluate group-by-time interaction effects on body composition (LM, FM, %BF), performance (VO_2_max, CMJ, 1RM strength), and blood variables. Sex differences were evaluated with a 3 × 2 repeated measures ANOVA with the between subject factor as sex. Simple main effects were evaluated by performing independent or paired samples t-test to compare treatment groups at each specific time point, using Bonferroni adjustments to account for multiple comparisons. Analyses were performed using SPSS (Version 27.0; IBM, Somers, NY, USA) with statistical significance set *a priori* at α = 0.05.

## Results

### Body composition

**FIGURE 3:**
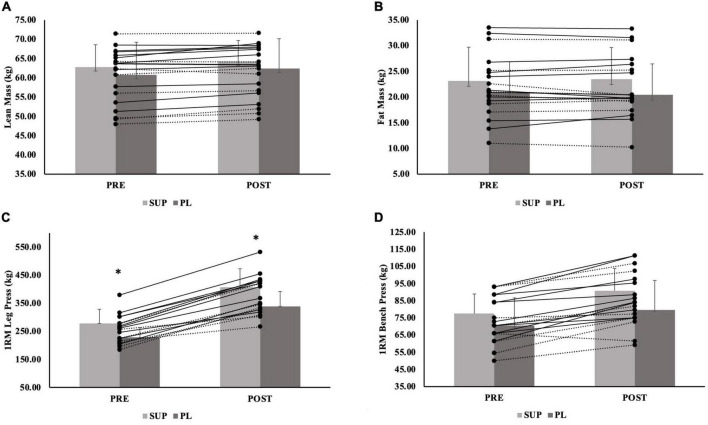
**(A–D)** Male individual responses for **(A)** lean mass, **(B)** fat mass, and **(C)** 1RM leg press, and **(D)** 1RM Bench Press. The lines represent the differences between baseline (PRE) and post-testing visit (POST) per participant. The solid lines represent males in the supplement group (*n* = 10). The dashed lines represent males in the PL group (*n* = 8). The gray bars represent treatment means for the supplement (SUP; light gray) and placebo (PL; dark gray) groups with average standard deviation (error bars). *Indicates significant main effect for group (*p* = 0.002).

**FIGURE 4:**
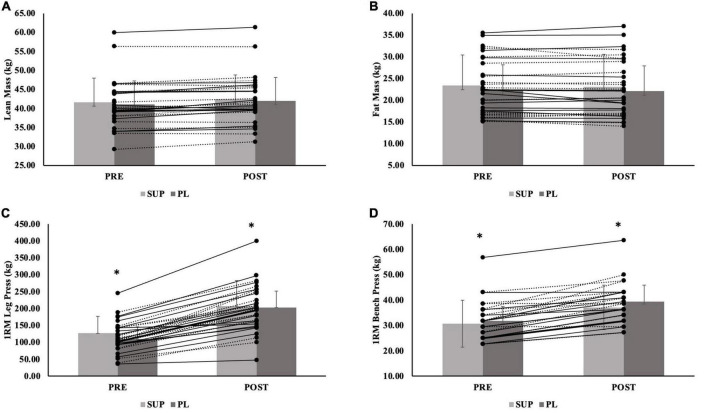
**(A–D)** Female individual responses for **(A)** lean mass, **(B)** fat mass, and **(C)** 1RM leg press, and **(D)** 1RM Bench Press. The lines represent the differences between baseline (PRE) and post-testing visit (POST) per participant. The gray bars represent treatment mean with average standard deviation. The solid lines represent females in the supplement group (*n* = 15). The dashed lines represent females in the placebo group (*n* = 16). The gray bars represent treatment means for the supplement (SUP; light gray) and placebo (PL; dark gray) groups with average standard deviation (error bars). *Indicates significant main effect for group for 1RM leg press (*p* = 0.004) and 1RM bench press (*p* = 0.035).

For total body mass, there was no significant group-by-time interaction (*p* = 0.124) or significant main effect for group (*p* = 0.302). There was a main effect for time {Mean difference [MD (Post-Pre)] ± Standard Error (SE) = −0.61 ± 0.22 kg; *p* = 004} (Table 3). For LM, there was no significant group-by-time interaction (*p* = 0.081) or significant main effect for group (*p* = 0.338). There was a main effect for time {Mean difference [MD (Post-Pre)] ± Standard Error (SE) = 0.78 ± 0.12 kg; *p* < 0.001}; the SUP group resulted in a greater increase in LM compared to the CON group (5.2 ± 3.8 kg; *p* = 0.510), although not significant. When separated by sex, there was no significant group-by-time interaction for males (*p* = 0.182) or females (*p* = 0.317). There was a main effect for time for males (0.84 ± 0.24 kg; *p* = 0.003) and females (0.73 ± 0.14 kg; *p* < 0.001). There was no main effect for group for males (*p* = 0.284) or females (*p* = 0.434). Individual effects for LM for males and females are presented in Figures 3, 4.

**TABLE 3:** Body composition, performance, and blood analyte variables presented as mean ± standard deviation.

**Body mass**			
	**Supplement**	**Placebo**	**Control**
Pre-intervention (kg)	77.2 ± 16.2	71.3 ± 12.3	71.4 ± 18.7
Post-intervention (kg)	78.4 ± 17.1	71.9 ± 12.3	71.5 ± 18.4
**Lean mass**			
	**Supplement**	**Placebo**	**Control**
Pre-intervention (kg)	50.1 ± 12.2	46.6 ± 10.6	45.2 ± 12.1
Post-intervention (kg)	51.2 ± 12.4	47.5 ± 10.3	45.6 ± 12.0
**Fat mass**			
	**Supplement**	**Placebo**	**Control**
Pre-intervention (kg)	24.3 ± 8.3	21.8 ± 5.9	23.4 ± 9.7
Post-intervention (kg)	24.2 ± 8.2	21.6 ± 5.9	23.2 ± 9.9
**Absolute VO_2_max**			
	**Supplement**	**Placebo**	**Control**
Pre-intervention (L/min)	2.9 ± 0.9	2.6 ± 0.9	2.4 ± 0.9
Post-intervention (L/min)	2.8 ± 0.9	2.6 ± 0.9	2.3 ± 0.8
**Relative VO_2_max**			
	**Supplement**	**Placebo**	**Control**
Pre-intervention (ml/kg/min)	37.3 ± 8.1	36.3 ± 8.3	34.0 ± 8.2
Post-intervention (ml/kg/min)	36.1 ± 7.2	35.3 ± 8.2	32.4 ± 8.0
Lower body strength*			
	**Supplement**	**Placebo**	**Control**
Pre-intervention (kg)	187.5 ± 89.9	149.7 ± 67.0	141.8 ± 105.5
Post-intervention (kg)	286.9 ± 121.9^†^	248.7 ± 81.9	152.9 ± 106.1^†^
**Upper body strength**			
	**Supplement**	**Placebo**	**Control**
Pre-intervention (kg)	49.4 ± 25.4	44.7 ± 21.4	43.9 ± 28.5
Post-intervention (kg)	58.6 ± 28.8	52.8 ± 22.2	43.1 ± 27.2
**Counter movement jump**			
	**Supplement**	**Placebo**	**Control**
Pre-intervention (cm)	34.6 ± 9.5	32.3 ± 8.7	30.4 ± 10.5
Post-intervention (cm)	35.6 ± 9.8	33.3 ± 7.6	30.9 ± 10.3
**Creatine kinase**			
	**Supplement (*n* = 22)**	**Placebo**	**Control**
Pre-intervention (ng/ml)	103.6 ± 48.5	96.7 ± 42.0	100.2 ± 59.4
Post-intervention (ng/ml)	108.9 ± 45.4	104.7 ± 35.5	123.1 ± 109.3
**Isoprostanes**			
	**Supplement**	**Placebo**	**Control**
Pre-intervention (ng/ml)	89.1 ± 32.6	85.0 ± 31.3	97.4 ± 34.5
Post-intervention (ng/ml)	92.1 ± 34.5	93.0 ± 31.6	104.0 ± 28.3

*Indicates significant main effect for group (*p* = 0.018). ^†^Indicates statistically significant difference between supplement and control group (*p* = 0.015) from *post-hoc* analysis.

For FM, there was no significant group-by-time interaction (*p* = 0.749), main effect for time (−0.19 ± 0.13 kg; *p* = 0.146), or main effect for group (*p* = 0.702). When separated by sex, there was no significant group-by-time interaction for males (*p* = 0.133) or females (*p* = 0.725), no main effect for time for males (0.29 ± 0.23 kg; *p* = 0.225) and females (0.18 ± 0.16 kg; *p* = 0.270), an no main effect for group for males (*p* = 0.643) or females (*p* = 0.925). Individual effects for FM for males and females are presented in Figures 3, 4.

### Performance

For absolute VO_2_max, there was no significant group-by-time interaction (*p* = 0.160), main effect for time (0.46 ± 0.31 L/min; *p* = 0.141), or main effect for group (*p* = 0.260). When separated by sex, there was no significant group-by-time interaction for males (*p* = 0.311) or females (*p* = 0.185), or main effect for time for males (0.05 ± 0.07 L/min; *p* = 0.522) or females (0.42 ± 0.03 L/min; *p* = 0.128). There was no main effect for group for males (*p* = 0.572) or females (*p* = 0.052). For females, the *post-hoc* analysis demonstrated a significant difference between the SUP and CON group (0.4 ± 0.2 L/min; *p* = 0.047).

For relative VO_2_max, there was no significant group-by-time interaction (*p* = 0.800). There was a significant main effect for time (−1.20 ± 0.46 mL/kg/min; *p* = 0.012), but no main effect for group (*p* = 0.374). VO_2_max decreased from baseline to post-training (−1.2 ± 0.5 mL/kg/min; *p* = 0.012). When separated by sex, there was no significant group-by-time interaction for males (*p* = 0.951) or females (*p* = 0.359), or main effect for time for males (−1.5 ± 1.10 mL/kg/min; *p* = 0.185). There was a main effect for time in females (−1.0 ± 0.42 mL/kg/min; *p* = 0.022). There was no main effect for group for males (*p* = 0.740) or females (*p* = 0.352).

For 1RM leg strength (LP1RM), there was a significant group-by-time interaction (*p* < 0.001). There was a main effect for time (69.8 ± 4.5 kg *p* < 0.001), and a main effect for group (*p* = 0.018); the SUP group gained significantly more strength than CON group (89.9 ± 30.8 kg; *p* = 0.015) (Table 3). The PL group also gained more 1RM leg strength than the CON group (51.9 ± 31.0 kg; *p* = 0.299), although not significant. When separated by sex, there was a significant group-by-time interaction for males (*p* < 0.001) and females (*p* < 0.001), main effect for time for males (82.9 ± 7.5 kg; *p* < 0.001) and females (61.6 ± 5.1 kg; *p* < 0.001), and main effect for group for males (*p* = 0.002) and females (*p* = 0.004). For males and females, the SUP group gained more strength than the CON group (149.7 ± 36.3 kg; *p* = 0.002 and 66.8 ± 19.6 kg; *p* = 0.005, respectively). For females, the PL group also gained more strength compared to the CON group (56.5 ± 19.4 kg; *p* = 0.018). Individual effects for LP1RM for males and females are presented in Figures 3, 4.

For 1RM upper body strength (BP1RM), there was a significant group-by-time interaction (*p* < 0.001) and main effect for time (5.5 ± 0.7 kg; *p* < 0.001), but no significant main effect for group (*p* = 0.439) (Table 3); the SUP group demonstrated greater increases than the CON group (10.5 ± 8.2 kg; *p* = 0.622), while not significant. When separated by sex, there was a significant group-by-time interaction for males (*p* = 0.002) and females (*p* < 0.001). There was a main effect for time for males (6.9 ± 1.5 kg; *p* < 0.001) and females (4.6 ± 0.6 kg; *p* < 0.001). There was no main effect for group for males (*p* = 0.450), but there was a main effect for females (*p* = 0.035). For females, the SUP group gained more strength compared to the CON group (6.6 ± 3.2 kg; *p* = 0.134), although not significant. The PL group gained more strength compared to the CON group (8.3 ± 3.1 kg; *p* = 0.035). Individual effects for BP1RM for males and females are presented in Figures 3, 4.

For CMJ, there was no significant group-by-time interaction (*p* = 0.886) (Table 3). There was a main effect for time (8.52 ± 0.40 cm; *p* = 0.040), but no main effect for group (*p* = 0.337). When separated by sex, there was no significant group-by-time interaction for males (*p* = 0.862) or females (*p* = 0.642). There was no main effect for time for males (−0.70 ± 5.5 cm; *p* = 0.899), but there was a main effect for time for females (1.4 ± 5.0 cm; *p* = 0.014). There was no main effect for group for males (*p* = 0.639) or females (*p* = 0.456).

### Blood analytes

For CK, there was no significant group-by-time interaction (*p* = 0.938), no main effect for time (−8.3 ± 6.7 ng/ml; *p* = 0.222), and no main effect for group (*p* = 0.703) (Table 3). When separated by sex, there was no significant group-by-time interaction for males (*p* = 0.651) or females (*p* = 0.650). There was no main effect for time for males (−0.2 ± 11.9 ng/ml; *p* = 0.987) or females (10.5 ± 8.4 ng/ml; *p* = 0.221). There was no main effect for group for males (*p* = 0.192) or females (*p* = 0.714).

For isoprostanes, there was no significant group-by-time interaction (*p* = 0.830) (Table 3). There was no main effect for time (5.88 ± 3.88 ng/ml; *p* = 0.135), and no main effect for group (*p* = 0.425). When separated by sex, there was no significant group-by-time interaction for males (*p* = 0.149) or females (*p* = 0.678), no main effect for time for males (0.7 ± 6.6 ng/ml; *p* = 0.916) or females (9.0 ± 4.7 ng/ml; *p* = 0.062), and no main effect for group for males (*p* = 0.587) or females (*p* = 0.575).

## Discussion

The current study evaluated a twice weekly 40-min HIRT + HIIT exercise paired with nutrition supplementation. These data were collected during the COVID-19 pandemic in 2020, which required a reduced number of training days for feasibility. In general, the COVID-19 pandemic emphasized the importance of identifying feasible exercise strategies that are time efficient (28). The present study demonstrated that a twice weekly 40-min concurrent HIRT + HIIT exercise intervention can support increases in LM and strength in men and women. There was a significantly greater improvement in upper and lower body strength when nutrients were consumed before and after exercise. Females appeared to respond more favorably to supplementation, with greater increases in upper and lower body strength. The combined HIRT + HIIT exercise may be a feasible option to improve strength and LM, with only two sessions per week for six weeks. These improvements were further augmented when nutrients were consumed before and after exercise, particularly for women.

Meta-analyses suggest that the stimulation of muscle protein synthesis and increases in LM are influenced by nutrient timing, particularly protein intake (17). There is conflicting information on the actual timing of nutrients, whether pre- or post-exercise is more advantageous (7, 15–18), and what time frame is required for nutrient consumption to support exercise adaptations (14, 18, 29). However, data collectively supports that consuming nutrients around exercise may provide greater benefit than withholding nutrients (17). The present study supports existing findings; LM increased significantly from baseline to post-testing with the SUP group gaining more LM (+1.1 kg) compared to the PL group (+0.88 kg) and CON group (+0.37 kg). The multi-ingredient formula consumed in the present study contained ingredients that have been known to support muscle protein synthesis (30) and tissue repair (20) [whey protein, casein protein, and β-hydroxy-β-methylbutyrate (HMB)], possibly supporting the greater, yet non-significant, increases in LM reported in the SUP group compared to CON. Furthermore, these increases in LM were observed without the addition of creatine monohydrate, a dietary supplement that has demonstrated improvements in LM especially when paired with resistance training (31). In a similar study evaluating nutrient timing around the same HIRT protocol implemented in women, consumption of nutrients (16 g CHO + 25 g PRO) before or after–exercise resulted in significantly greater increases in LM compared to no nutritional intake (PRE: +0.96 kg; Post: +0.64 kg; CON: +0.15 kg) (7). The present study resulted in similar improvements in LM in females (SUP: +0.9 kg; PL: +0.9 kg; CON: +0.4 kg). It is well-known that resistance training increases LM (32, 33), with an expected +2.8% gain in LM post 6-weeks of HIRT (10). Uniquely, in addition to HIRT, the present study included a concurrent aerobic HIIT bout, which has demonstrated increases in LM in as little as three weeks when performed by itself (6). Concurrent training with resistance training and HIIT has been suggested as an effective method for maintaining strength and LM (13). In support, the present study demonstrated a +2.2% gain in LM from baseline values within the SUP group, as well as a +1.9% gain within the PL group from baseline when concurrent training was employed. It appears that when HIRT is performed prior to HIIT, increases in LM and strength may result over time, particularly when nutrients are provided around the exercise.

Prior research has demonstrated that HIRT and HIIT elicit reductions in FM, possibly through increased fatty acids utilization during exercise (34, 35). However, studies including nutrient consumption surrounding high-intensity exercise have not demonstrated augmented FM loss (7, 8), most likely due to the lack of diet modification. In the present study, there were no changes in FM across time (−0.19 kg) or between groups. The results are consistent with previous studies utilizing similar exercise protocols, with or without nutritional supplementation, which have reported decreases in FM post-intervention ranging from −0.1 to −0.6 kg (8, 10, 36). The lack of significant changes in FM is primarily due to the absence of day-to-day dietary control. Fat loss with exercise is often not pronounced without caloric restriction or dietary intervention. The present study was not aimed at reducing calories or changing dietary intake, but rather providing specific nutrients surrounding a time effective exercise session. In contrast, the present study provided 220 calories twice per week with the pre- and post-supplementation, an amount that did not appear to influence body weight or FM. The nutritional supplement utilized in the present study included ingredients like caffeine and whey protein, which have previously supported FM loss while sparing LM during a caloric deficit (37, 38). Twice weekly supplementation, as well as the lack of planned caloric restriction likely impacted the lack of FM loss in the present study.

It is well-known that traditional RT provides a potent anabolic stimulus, which can result in increased muscle strength and maintenance of LM (39). Data collectively supports that RT improves muscle strength, quality, and may assist in prevention of chronic diseases (40). However, lack of time is often cited as a major barrier for participation in RT exercises (41). HIRT is a time effective RT approach that has demonstrated significant and rapid improvements in maximal strength despite the relatively short time of effort (7, 10). Despite the short rest period between sets, participants were able to successfully complete 6–8 repetitions in each set. The present study supports existing findings with HIRT (7, 10, 13) demonstrating improvements in lower body strength baseline to post-testing in the SUP group (+99.4 kg) and PL group (+99.0 kg), compared to the CON group (+11.1 kg). While not significant, the SUP group (+9.2 kg) and PL group (+8.1 kg) demonstrated greater upper body strength increases compared to the and CON group (−0.8 kg). The HIRT intervention may be beneficial for increases in strength. Additionally, consumption of nutrients before and after exercise, particularly, protein, carbohydrates, and caffeine, may have provided a greater environment to support muscular adaptations associated with HIRT (42–44). A recent study evaluating six-weeks of HIRT training in healthy males and females resulted in significantly greater increases in lower (LP1RM: +49.9 kg) and upper body 1RM (BP1RM: +11.4 kg) strength when compared to traditional resistance training (LP1RM: +31.6 kg; BP1RM −7.9 kg), highlighting the impact of a short-term whole-body HIRT training approach for increasing strength in males and females. Existing data suggest that males and females respond similarly when beginning RT (45), yet females potentially experience greater increases in strength (46). Our findings support a potential sexually dimorphic response, with females in both the SUP and PL group significantly increasing LP1RM and BP1RM when compared to CON, while only males in the SUP group saw significant improvements in strength. As such, future research should explore sex differences in nutrient timing and HIRT adaptations, particularly as data in males cannot always be extrapolated to females.

In addition to maximal strength, the present study evaluated other performance outcomes such as aerobic fitness *via* VO_2_max and lower body power *via* CMJ. HIIT training has been shown to augment cardiorespiratory fitness with data demonstrating VO_2_max improvements in as little as six weeks (4, 47). In contrast to previous research, there was no change in absolute VO_2_max values following the intervention, which is possibly due to the concurrent nature of the HIRT + HIIT on molecular pathways, with the HIIT taking place immediately after the HIRT exercises. Participants may have been fatigued from the HIRT session thereby limiting cardiorespiratory adaptations to HIIT. Additionally, due to the intense nature of the intervention, participants may not have fully recovered prior to when the post-testing was completed (∼48 h of the last training session). In addition to cardiorespiratory improvements, high-intensity exercise, whether aerobic or anaerobic, requires high locomotor speed and power (48). Maximal CMJ is a strong assessment of lower-body power test with good reliability (intraclass correlation, >0.989). There are limited data regarding the effects of HIRT and HIIT on CMJ (49, 50); the present study demonstrated positive effects of combined high-intensity strength and interval training on CMJ.

Creatine kinase and isoprostanes have consistently been used as indicators of muscle damage and oxidative stress, respectively (51, 52). It has been postulated that consumption of nutrients such as carbohydrate and protein surrounding high-intensity exercise may attenuate markers of muscle damage and oxidative stress (53, 54). While some research reports that protein-carbohydrate supplementation surrounding high-intensity exercise significantly reduces post-exercise serum CK levels (54, 55), other studies have not (56, 57). In the present study, there were no significant changes in CK across time or between groups. It remains unclear whether consumption of nutrients before or after high-intensity exercise mitigates markers of muscle damage (58). While the time of day was consistent between the baseline and post-testing visit, there may have been some variability in the post-testing values due to the difference in testing days after the last exercise session. The magnitude of oxidative stress following an acute bout of exercise is generally proportional to exercise intensity (59). However, there is limited research evaluating markers of oxidative stress, such as isoprostanes, after HIRT or the influence of nutrient timing on isoprostanes levels post-exercise. One previous study observing carbohydrate ingestion during RT in males reported acute isoprostanes levels were unaffected (53). Our findings may support this as there were no changes in isoprostanes across time or between groups. It appears that blood markers were not significantly impacted by nutrient timing in the present study.

The results of the present study reflect the impact of a minimal nutritional intervention with exercise, resulting in significant increases in LM and strength; nutrient consumption outside of the pre- and post-workout supplementation was not modified or monitored throughout the study. Participants’ relative protein intake (g/kg) was lower (Table 1) than the recommended amount for increasing LM (1.6–2.0 g/kg) (17), and the addition of the 20 grams of protein twice a week would have only increased protein intake to 1.3 g/kg on the training days. A larger change in calorie and macronutrient composition, on more than two days per week, would have likely supported more pronounced effects on body composition. Additionally, some of the components of the multi-ingredient pre-workout formulation (e.g., caffeine, HMB, and carbohydrates) in the present study may have been lower compared to the recommended dosing amount (17). Future research may benefit from exploring a relative dose of the individual supplements vs. an absolute dose product as utilized in the present study. Although weight loss was not a primary outcome of the present study, results suggest future research targeting weight loss and FM loss may benefit from dietary modification in addition to nutrient supplementation and HIRT. The investigation of sex differences in body composition and performance was an exploratory aim of the present study. The sample size of males and females in each group may need to be larger in future studies. Additionally, as a result of extensive research cleaning and spacing restrictions during the COVID-19 pandemic, the present intervention was only conducted on two days per week; inclusion of an additional day may have resulted in greater adaptations.

## Conclusion

In conclusion, the twice weekly nutrition supplementation before and after the high-intensity exercise protocol appears to be an effective approach for increasing LM and strength, especially in females. These effects are likely attributed to nutrients supporting muscle recovery and providing anabolic stimuli for muscle growth in response to HIRT + HIIT (14). Females appeared to respond more favorably to nutrient consumption, demonstrating greater increases in upper and lower body strength. Future research should continue to explore sex differences in nutrient timing and HIRT adaptations, particularly as data in males cannot always be generalized to females. The present study suggests there are beneficial effects of the exercise and nutrition intervention despite minimal training time and lifestyle changes.

## Data availability statement

Deidentified individual data that support the results will be shared upon a reasonable request beginning 12–36 months following publication provided the investigator who proposes to use the data has approval from an Institutional Review Board (IRB), Independent Ethics Committee (IEC), or Research Ethics Board (REB), as applicable, and executes a data use/sharing agreement with UNC. Requests to access the datasets should be directed to abbsmith@email.unc.edu.

## Ethics statement

The studies involving human participants were reviewed and approved by the University of North Carolina Institutional Review Board. The patients/participants provided their written informed consent to participate in this study.

## Author contributions

AS-R designed the study. HC, AG, NP, and A-SR collected the data and contributed to the manuscript preparation. HC and AS-R analyzed and interpreted the data. All authors reviewed and approved final manuscript.
